# Supplementary material for: Analysis of QTL mapping for germination and seedling response to drought stress in sunflower (Helianthus annuus L.)
Source: PeerJ. 2023 May 3;11:e15275. doi: 10.7717/peerj.15275 (PMC10163870; doi:10.7717/peerj.15275)
Supplement: Supplemental Information 2 — ** Correlation is significant at the 0.01 level. * Correlation is significant at the 0.05 level. The upper right corner of the table represents the correlation coefficients between traits under drought stress; the lower left corner of the table represents the correlation coefficients between traits under normal conditions. [file peerj-11-15275-s002.docx]

|  | **Supplementary Table 1. phenotypic correlation between traits measured under control and drought conditions** | | | | | | | | | | | | | | | | | | |
| --- | --- | --- | --- | --- | --- | --- | --- | --- | --- | --- | --- | --- | --- | --- | --- | --- | --- | --- | --- |
|  | **DS** | | | | | | | | | | | | | | | | | | |
|  |  | **GL** | **RL** | **GFW** | **GDW** | **RFW** | **RDW** | **GP** | **GR** | **GI** | **RSA** | **RSR** | **RLS** | **RLWC** | **PHS** | **GFWS** | **UFWS** | **GDWS** | **UDWS** |
| **CK** | **GL** |  | 0.613** | 0.637** | 0.275** | 0.631** | 0.484** | 0.405** | 0.316** | 0.355** | -0.001 | -0.112 | 0.068 | -0.116 | 0.212** | 0.091 | 0.085 | 0.112 | -0.02 |
|  | **RL** | 0.701** |  | 0.649** | 0.350** | 0.734** | 0.655** | 0.570** | 0.483** | 0.505** | -0.018 | -0.06 | 0.169* | -0.051 | 0.132 | -0.037 | -0.103 | 0.097 | 0.057 |
|  | **GFW** | 0.624** | 0.689** |  | 0.609** | 0.833** | 0.676** | 0.601** | 0.475** | 0.515** | -0.06 | -0.12 | 0.055 | -0.056 | 0.094 | 0.061 | 0.016 | 0.151 | 0.085 |
|  | **GDW** | 0.023 | 0.121 | 0.539** |  | 0.478** | 0.447** | 0.473** | 0.361** | 0.370** | -0.056 | -0.057 | 0.083 | -0.113 | -0.071 | -0.008 | -0.002 | 0.067 | 0.016 |
|  | **RFW** | 0.489** | 0.739** | 0.769** | 0.279** |  | 0.746** | 0.499** | 0.393** | 0.428** | -0.036 | -0.086 | 0.134 | -0.076 | 0.109 | -0.006 | -0.056 | 0.097 | 0.051 |
|  | **RDW** | 0.360** | 0.632** | 0.661** | 0.403** | 0.730** |  | 0.466** | 0.346** | 0.342** | -0.066 | -0.12 | 0.064 | -0.006 | 0.073 | 0.059 | -0.113 | 0.135 | 0.008 |
|  | **GP** | 0.431** | 0.421** | 0.438** | 0.223** | 0.361** | 0.361** |  | 0.919** | 0.921** | -0.106 | -0.109 | 0.007 | -0.205* | 0.111 | 0.045 | 0.036 | 0.043 | -0.028 |
|  | **GR** | 0.403** | 0.375** | 0.359** | 0.192* | 0.289** | 0.300** | 0.977** |  | 0.956** | -0.079 | -0.045 | 0.011 | -0.196* | 0.049 | 0.041 | 0.046 | 0.002 | -0.011 |
|  | **GI** | 0.376** | 0.360** | 0.321** | 0.174* | 0.253** | 0.265** | 0.927** | 0.950** |  | -0.099 | -0.063 | 0.001 | -0.195* | 0.029 | 0.03 | 0.045 | -0.017 | -0.025 |
|  | **RSA** | 0.012 | 0.028 | 0.062 | 0.014 | 0.049 | 0.102 | 0.067 | 0.067 | 0.085 |  | 0.323** | 0.682** | 0.176* | 0.115 | -0.127 | -0.026 | 0.154 | 0.231** |
|  | **RSR** | -0.04 | 0.095 | 0.018 | -0.053 | 0.109 | 0.064 | -0.06 | -0.062 | -0.07 | 0.042 |  | 0.144 | 0.002 | -0.252** | -0.220** | 0.056 | -0.346** | 0.467** |
|  | **RLS** | 0.048 | 0.106 | 0.026 | -0.063 | 0.034 | 0.116 | 0.069 | 0.068 | 0.051 | 0.552** | 0.103 |  | 0.039 | 0.112 | -0.032 | 0.026 | 0.279** | 0.204* |
|  | **RLWC** | 0.159 | 0.139 | 0.119 | 0.025 | 0.095 | 0.158 | 0.025 | 0.02 | 0.032 | 0.055 | -0.044 | -0.159 |  | 0.078 | -0.037 | -0.038 | -0.015 | -0.071 |
|  | **PHS** | 0.187* | 0.002 | 0.087 | -0.065 | 0.016 | -0.055 | 0.187* | 0.168* | 0.121 | 0.06 | -0.027 | 0.102 | -0.108 |  | 0.337** | 0.03 | 0.443** | 0.145 |
|  | **GFWS** | 0.029 | 0.059 | 0.125 | 0.14 | 0.078 | 0.126 | -0.007 | -0.007 | -0.015 | 0.276** | -0.103 | 0.055 | 0.098 | 0.163* |  | 0.420** | 0.317** | 0.141 |
|  | **UFWS** | 0.214** | 0.402** | 0.474** | 0.281** | 0.617** | 0.471** | 0.088 | 0.055 | 0.02 | 0.347** | 0.216** | 0.207* | 0.003 | 0.091 | 0.231** |  | -0.109 | 0.018 |
|  | **GDWS** | 0.123 | 0.025 | 0.109 | 0.036 | 0.081 | 0.092 | 0.029 | 0.021 | 0.034 | 0.151 | -0.358** | -0.002 | -0.055 | 0.241** | 0.382** | 0.134 |  | 0.496** |
|  | **UDWS** | 0.029 | 0.168* | 0.229** | 0.097 | 0.287** | 0.383** | -0.044 | -0.063 | -0.084 | 0.04 | 0.523** | 0.132 | -0.112 | 0.101 | 0.107 | 0.381** | 0.251** |  |
