# Supplementary material for: Analysis of QTL mapping for germination and seedling response to drought stress in sunflower (Helianthus annuus L.)
Source: PeerJ. 2023 May 3;11:e15275. doi: 10.7717/peerj.15275 (PMC10163870; doi:10.7717/peerj.15275)
Supplement: Supplemental Information 3 [file peerj-11-15275-s003.docx]

**Supplementary Table 2. A total of 60 candidate genes were screened from a RIL population at two periods**

| **QTL** | **Gene ID** | **chr** | **start.x** | **end.x** | **COG_class** | **COG_class_annotation** | **GO_annotation** | **KEGG_annotation** | **nr_annotation** |
| --- | --- | --- | --- | --- | --- | --- | --- | --- | --- |
| **qGFW-3-1** | LOC110930297 | 3 | 139179499 | 139188527 | -- | -- | -- | K09286\|4.00373e-40\|spen:107012057\|K09286 EREBP-like factor \| (RefSeq) ethylene-responsive transcription factor RAP2-3 | ethylene-responsive transcription factor RAP2-3-like [Helianthus annuus] |
| **qUFWSn-4-1** | LOC110938147 | 4 | 175842854 | 175843300 | [QV] | -- | -- | K10528\|0\|fve:101298061\|K10528 hydroperoxide lyase [EC:4.1.2.-] \| (RefSeq) linolenate hydroperoxide lyase, chloroplastic | putative cytochrome P450 [Helianthus annuus] |
| **qUFWSn-4-1** | LOC110938090 | 4 | 173952597 | 173953481 | [QV] | -- | -- | K07408\|2.8219e-147\|pxb:103926936\|K07408 cytochrome P450 family 1 subfamily A polypeptide 1 [EC:1.14.14.1] \| (RefSeq) cytochrome P450 71A3-like | cytochrome P450 71BL1-like [Helianthus annuus] |
| **qUFWSn-4-1** | LOC110938156 | 4 | 176067711 | 176067935 | -- | -- | -- | -- | WRKY transcription factor 6-like [Helianthus annuus] |
| **qGFW-6-1** | LOC110864658 | 6 | 12214793 | 12227602 | [V] | Defense mechanisms | -- | -- | ABC transporter G family member 35-like [Helianthus annuus] |
| **qGFW-6-1** | LOC110865034 | 6 | 26478912 | 26482020 | [QV] | -- | -- | K09843\|0\|sind:105157462\|K09843 (+)-abscisic acid 8'-hydroxylase [EC:1.14.13.93] \| (RefSeq) abscisic acid 8'-hydroxylase 1-like | abscisic acid 8&apos;-hydroxylase 1-like isoform X1 [Helianthus annuus] |
| **qGFW-6-1** | LOC110864775 | 6 | 15942406 | 15944019 | [QV] | -- | -- | K15398\|0\|sind:105172724\|K15398 fatty acid omega-hydroxylase [EC:1.14.-.-] \| (RefSeq) cytochrome P450 86A8-like | cytochrome P450 86A8-like [Helianthus annuus] |
| **qGFW-6-1** | LOC110864791 | 6 | 16559552 | 16561178 | -- | -- | -- | -- | putative transcription factor GRAS [Helianthus annuus] |
| **qGDWSn-7-1** | LOC110867995 | 7 | 33744133 | 33745068 | -- | -- | -- | -- | ethylene-responsive transcription factor CRF2-like [Helianthus annuus] |
| **qGDWSn-7-1** | LOC110869161 | 7 | 98748532 | 98749467 | -- | -- | -- | K09286\|1.47616e-43\|sind:105179200\|K09286 EREBP-like factor \| (RefSeq) ethylene-responsive transcription factor 5-like | ethylene-responsive transcription factor 5-like [Helianthus annuus] |
| **qGDWSn-7-1** | LOC110869294 | 7 | 102715918 | 102717072 | -- | -- | -- | -- | ethylene-responsive transcription factor CRF4-like [Helianthus annuus] |
| **qGDWSn-7-1** | LOC110866308 | 7 | 38367555 | 38368163 | -- | -- | -- | -- | ethylene-responsive transcription factor ERF109-like [Helianthus annuus] |
| **qGDWSn-7-1** | LOC110868808 | 7 | 84306489 | 84307382 | -- | -- | -- | K09284\|5.00501e-90\|csv:101222024\|K09284 AP2-like factor, euAP2 lineage \| (RefSeq) ethylene-responsive transcription factor RAP2-7 | AP2-like ethylene-responsive transcription factor TOE3 isoform X1 [Helianthus annuus] |
| **qGDWSn-7-1** | LOC110868679 | 7 | 78560202 | 78560486 | -- | -- | -- | K09286\|1.00121e-46\|gra:105777318\|K09286 EREBP-like factor \| (RefSeq) ethylene-responsive transcription factor RAP2-3 | ethylene-responsive transcription factor RAP2-3-like [Helianthus annuus] |
| **qGDWSn-7-1** | LOC110869265 | 7 | 101678634 | 101679109 | -- | -- | -- | -- | ethylene-responsive transcription factor ERF027-like [Helianthus annuus] |
| **qGDWSn-7-1** | LOC110868376 | 7 | 63775921 | 63776015 | -- | -- | -- | -- | WRKY transcription factor 44-like [Helianthus annuus] |
| **qGDWSn-7-1** | LOC110868391 | 7 | 64766264 | 64767279 | [QV] | -- | -- | -- | cytochrome P450 71B34-like [Helianthus annuus] |
| **qGDWSn-7-1** | LOC110868886 | 7 | 86563450 | 86563832 | -- | -- | -- | K03327\|1.4846e-34\|sind:105155759\|K03327 multidrug resistance protein, MATE family \| (RefSeq) protein TRANSPARENT TESTA 12-like | putative cytochrome P450 [Helianthus annuus] |
| **qGDWSn-7-1** | LOC110867922 | 7 | 27327408 | 27327792 | [QV] | -- | -- | -- | cytochrome P450 CYP72A219-like [Helianthus annuus] |
| **qGDWSn-7-1** | LOC110867927 | 7 | 27592946 | 27593225 | [QV] | -- | -- | -- | cytochrome P450 CYP72A219-like [Helianthus annuus] |
| **qGDWSn-7-1** | LOC110868163 | 7 | 49858536 | 49860068 | [QV] | -- | -- | K20665\|0\|vvi:100245827\|K20665 jasmonoyl-isoleucine 12-hydroxylase [EC:1.14.-.-] \| (RefSeq) cytochrome P450 94B3 | cytochrome P450 94B3-like [Helianthus annuus] |
| **qGDWSn-7-1** | LOC110867138 | 7 | 95327580 | 95327859 | [QV] | -- | -- | -- | cytochrome P450 CYP72A219-like [Helianthus annuus] |
| **qGDWSn-7-1** | LOC110869089 | 7 | 95397310 | 95397671 | [QV] | -- | -- | -- | cytochrome P450 CYP72A219-like [Helianthus annuus] |
| **qGDWSn-7-1** | LOC110869248 | 7 | 101431567 | 101432005 | [QV] | -- | -- | -- | cytochrome P450 704C1-like isoform X3 [Helianthus annuus] |
| **qGDWSn-7-1** | LOC110869088 | 7 | 95361094 | 95361491 | [QV] | -- | -- | -- | cytochrome P450 CYP72A219-like [Helianthus annuus] |
| **qGDWSn-7-1** | LOC110868317 | 7 | 61564064 | 61564480 | [G] | Carbohydrate transport and metabolism | -- | K09872\|2.30306e-179\|rcu:8284532\|K09872 aquaporin PIP \| (RefSeq) Pip2-2; probable aquaporin PIP2-2 | aquaporin PIP2-4-like [Helianthus annuus] |
| **qGDWSn-7-1** | LOC110868334 | 7 | 61870895 | 61871267 | [G] | Carbohydrate transport and metabolism | -- | K09872\|0\|rcu:8284532\|K09872 aquaporin PIP \| (RefSeq) Pip2-2; probable aquaporin PIP2-2 | aquaporin PIP2-1-like [Helianthus annuus] |
| **qGDWSn-7-1** | LOC110868358 | 7 | 63083718 | 63084122 | [G] | Carbohydrate transport and metabolism | -- | K09872\|0\|rcu:8284532\|K09872 aquaporin PIP \| (RefSeq) Pip2-2; probable aquaporin PIP2-2 | aquaporin PIP2-4-like [Helianthus annuus] |
| **qGDWSn-7-1** | LOC110868335 | 7 | 61838715 | 61839039 | [G] | Carbohydrate transport and metabolism | -- | K09872\|0\|rcu:8284532\|K09872 aquaporin PIP \| (RefSeq) Pip2-2; probable aquaporin PIP2-2 | aquaporin PIP2-4-like [Helianthus annuus] |
| **qGDWSn-7-1** | LOC110868941 | 7 | 88604562 | 88604972 | [G] | Carbohydrate transport and metabolism | -- | K09872\|0\|jcu:105646538\|K09872 aquaporin PIP \| (RefSeq) PIP1; probable aquaporin PIP-type 7a | probable aquaporin PIP1-4 [Helianthus annuus] |
| **qGDWSn-7-1** | LOC110868746 | 7 | 81526102 | 81527526 | -- | -- | -- | -- | proline-rich receptor-like protein kinase PERK8 [Helianthus annuus] |
| **qGDWSn-7-1** | LOC110869276 | 7 | 102226829 | 102227198 | [J] | Translation, ribosomal structure and biogenesis | -- | K01881\|0\|sind:105166697\|K01881 prolyl-tRNA synthetase [EC:6.1.1.15] \| (RefSeq) proline--tRNA ligase | proline--tRNA ligase, chloroplastic/mitochondrial-like [Helianthus annuus] |
| **qGDWSn-7-1** | LOC110868885 | 7 | 86535763 | 86536224 | [O] | Posttranslational modification, protein turnover, chaperones | -- | K13993\|5.25633e-63\|sind:105160926\|K13993 HSP20 family protein \| (RefSeq) 17.6 kDa class I heat shock protein-like | heat shock protein 17 [Helianthus annuus] |
| **qGDWSn-7-1** | LOC110869013 | 7 | 91043274 | 91043903 | -- | -- | -- | K14496\|7.139e-101\|sot:102580526\|K14496 abscisic acid receptor PYR/PYL family \| (RefSeq) abscisic acid receptor PYL4-like | abscisic acid receptor PYL4-like [Helianthus annuus] |
| **qGDWSn-7-1** | LOC110867930 | 7 | 28341847 | 28342177 | -- | -- | -- | -- | dehydrin Xero 1-like [Helianthus annuus] |
| **qGDWSn-7-1** | LOC110868160 | 7 | 49630027 | 49630274 | -- | -- | -- | -- | GATA transcription factor 12-like [Helianthus annuus] |
| **qGDWSn-7-1** | LOC110869179 | 7 | 99394240 | 99394917 | -- | -- | -- | -- | peroxisomal membrane protein 11-4-like [Helianthus annuus] |
| **qGFW-8-1** | LOC110873218 | 8 | 95688105 | 95691332 | -- | -- | -- | K13424\|6.0205e-171\|vvi:100243352\|K13424 WRKY transcription factor 33 \| (RefSeq) WRKY transcription factor WRKY24 | probable WRKY transcription factor 26 [Helianthus annuus] |
| **qGRn,**  **GPn,**  **GIn-9-1** | LOC110877496 | 9 | 47634214 | 47641700 | -- | -- | NA | -- | probable WRKY transcription factor 32 [Helianthus annuus] |
| **qGRn,**  **GPn,**  **GIn-9-1** | LOC110877508 | 9 | 48245841 | 48253530 | [P] | Inorganic ion transport and metabolism | NA | K00327\|0\|vvi:100240979\|K00327 NADPH-ferrihemoprotein reductase [EC:1.6.2.4] \| (RefSeq) NADPH--cytochrome P450 reductase | NADPH--cytochrome P450 reductase-like [Helianthus annuus] |
| **qRSAn,**  **qGFWSn-10-1** | LOC110886051 | 10 | 199211308 | 199211797 | -- | -- | -- | -- | probable dehydrin LEA [Helianthus annuus] |
| **qRSAn,**  **qGFWSn-10-1** | LOC110886021 | 10 | 197013387 | 197013720 | -- | -- | -- | -- | WRKY transcription factor 6-like [Helianthus annuus] |
| **qGDW,**  **qRSRn-13-1** | LOC110898128 | 13 | 76176966 | 76177337 | -- | -- | -- | K09875\|1.05629e-97\|vvi:100232979\|K09875 aquaporin SIP \| (RefSeq) small basic intrinsic protein 1 | aquaporin SIP1-2-like [Helianthus annuus] |
| **qGDW-13-1** | LOC110902688 | 13 | 57681562 | 57682805 | [O] | Posttranslational modification, protein turnover, chaperones | -- | K00799\|1.16268e-97\|fve:101294657\|K00799 glutathione S-transferase [EC:2.5.1.18] \| (RefSeq) glutathione S-transferase T1 | glutathione S-transferase T1-like [Helianthus annuus] |
| **qGDW-13-1** | LOC110897853 | 13 | 52109758 | 52111610 | [O] | Posttranslational modification, protein turnover, chaperones | -- | K00799\|3.6523e-102\|fve:101294657\|K00799 glutathione S-transferase [EC:2.5.1.18] \| (RefSeq) glutathione S-transferase T1 | glutathione S-transferase T1-like [Helianthus annuus] |
| **qGDW,**  **qRSRn,**  **qUDWS-13-1** | LOC110898092 | 13 | 74380804 | 74382288 | [QV] | -- | -- | -- | cytochrome P450 94C1-like [Helianthus annuus] |
| **qGDW,**  **qUDWS-13-1** | LOC110898071 | 13 | 72414336 | 72414506 | -- | -- | -- | -- | GABA transporter 1-like isoform X2 [Helianthus annuus] |
| **qGDW-13-1** | LOC110902670 | 13 | 54582547 | 54583218 | -- | -- | -- | -- | late embryogenesis abundant protein At1g64065-like [Helianthus annuus] |
| **qGDW-13-1** | LOC110902674 | 13 | 54832827 | 54833333 | -- | -- | -- | -- | late embryogenesis abundant protein At1g64065-like [Helianthus annuus] |
| **qGDW,**  **qUDWS-13-1** | LOC110898072 | 13 | 72430086 | 72430319 | -- | -- | -- | -- | GABA transporter 1-like [Helianthus annuus] |
| **qRSRn,**  **qUDWS-13-1** | LOC110898325 | 13 | 86174090 | 86174389 | [QV] | -- | -- | K20618\|0\|pop:POPTR_0006s09580g\|K20618 cytochrome P450 family 76 subfamily A \| (RefSeq) POPTRDRAFT_560858; hypothetical protein | putative cytochrome P450 [Helianthus annuus] |
| **qRSRn-13-1** | LOC110898429 | 13 | 91336775 | 91337145 | [V] | Defense mechanisms | -- | -- | ABC transporter G family member 15-like [Helianthus annuus] |
| **qRSRn,**  **qUDWS-13-1** | LOC110898478 | 13 | 94275495 | 94276232 | -- | -- | -- | K09286\|3.43261e-67\|nnu:104592095\|K09286 EREBP-like factor \| (RefSeq) dehydration-responsive element-binding protein 3-like | ethylene-responsive transcription factor TINY-like [Helianthus annuus] |
| **qRSRn, qUDWS-13-1** | LOC110898466 | 13 | 93416457 | 93416674 | [G] | Carbohydrate transport and metabolism | -- | K09873\|1.23494e-98\|sind:105164750\|K09873 aquaporin TIP \| (RefSeq) aquaporin TIP4-1 | aquaporin TIP4-1 [Helianthus annuus] |
| **qRSRn, qUDWS-13-1** | LOC110898141 | 13 | 76934355 | 76936842 | -- | -- | -- | -- | NAC domain-containing protein 100-like [Helianthus annuus] |
| **qRSRn, qUDWS-13-1** | LOC110898323 | 13 | 85776090 | 85780064 | [T] | Signal transduction mechanisms |  |  | probable serine/threonine-protein kinase PBL19 |
| **qRSRn-13-1** | LOC110898546 | 13 | 97456619 | 97456812 | -- | -- | -- | K00434\|9.88977e-135\|vvi:100247405\|K00434 L-ascorbate peroxidase [EC:1.11.1.11] \| (RefSeq) L-ascorbate peroxidase-like | putative L-ascorbate peroxidase 6 [Helianthus annuus] |
| **qUDWS-13-1** | LOC110898274 | 13 | 83335089 | 83337263 | [T] | Signal transduction mechanisms | -- | -- | ABC transporter G family member 20-like [Helianthus annuus] |
| **qRFWn-14-1** | LOC110908665 | 14 | 81132808 | 81141283 | [QV] | -- | -- | K20667\|0\|sind:105170203\|K20667 beta-amyrin 28-monooxygenase [EC:1.14.13.201] \| (RefSeq) beta-amyrin 28-oxidase-like | putative cytochrome P450 [Helianthus annuus] |
| **qRFWn-14-1** | LOC110908584 | 14 | 73597898 | 73598797 | -- | -- | -- | -- | ethylene-responsive transcription factor ERF062-like [Helianthus annuus] |
